# A Dual-Barrel Carbon Fiber Microelectrode for Generator–Collector Experiments

**DOI:** 10.1021/acsomega.5c05321

**Published:** 2025-08-07

**Authors:** Paula C. Falcoswki, Douglas P. M. Saraiva, Nicolas A. Ishiki, Edson A. Ticianelli, Mauro Bertotti

**Affiliations:** † Departamento de Química Fundamental, Instituto de Química, 28133 Universidade de São Paulo, São Paulo 05508-900, Brazil; ‡ Université Paris Cité, ITODYS, CNRS, Paris 75006, France; § Departamento de Físico Química, Instituto de Química de São Carlos, Universidade de São Paulo C.P. 780, São Carlos 13560-970, Brazil

## Abstract

Generator–collector systems have been intrinsically related to electrochemical studies for decades, from kinetics studies to quantification processes. Herein, a new protocol for fabricating a dual carbon fiber microelectrode using a dual-barrel capillary is described. The fabrication process involves the use of inexpensive carbon fiber and requires minimal specialized equipment, except for a micropipette puller. The resulting microelectrodes exhibit a microdisc geometry, which is advantageous due to their well-defined diffusion profiles. The electrode was characterized electrochemically and through imaging experiments (scanning electron microscopy). The device was then employed in a generator–collector mode, and using [Ru­(NH_3_)_6_]­Cl_3_ as a redox probe in an SECM configuration, an increase in the collection efficiency was observed during the approach to an insulating substrate. The dual carbon fiber microelectrode was also explored to obtain analytical information based on the correlation between the collection efficiency in the iodide/iodine system and the thiosulfate concentration.

## Graphical abstract

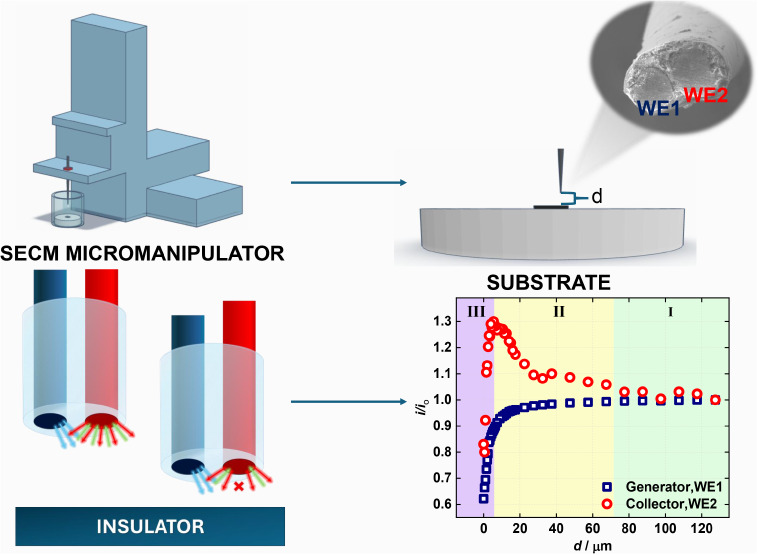

## Introduction

Over the years, various generator–collector systems have been extensively studied in the literature due to their wide range of applications. These systems are used in investigations of processes involving the dissolution of the generator electrode,^1^ elucidation of electrochemical reaction mechanisms,^2^ determination of kinetic constants,^3^ indirect detection of electroactive or nonelectroactive analytes,^4,5^ determination of diffusion coefficients in different media,^6,7^ and ionic transport between distinct phases,^8,9^ among other applications.

These systems are characterized by at least two distinct working electrodes: the generator electrode, responsible for the electrochemical production of a species, and the collector electrode, which collects this species or one of its derivatives. The transport of the species between the electrodes can occur by convection or diffusion, and different approaches for this have been explored in the literature, including flow systems,^10^ rotating ring-disk electrodes (RRDE),^11^ cavity transport,^12^ and dual microelectrode devices.^13–17^ Furthermore, these devices can be helpful in indirect measurements of nonelectroactive molecules, which react with some species produced in the generator electrode, decreasing, proportionally to the analyte concentration, the collection efficiency^18^ or changing the local pH that favors some electrochemical reactions.^19,20^

In stationary solutions (no forced convection), it is essential that the electrodes are positioned at a minimal distance from each other,^21^ on the order of micrometers, to enhance the sensitivity. This proximity can be achieved by designing two electrodes within the body of a split capillary, commonly called theta.^13,22–25^ A device with two electrodes in a generator–collector system is particularly useful for determining and quantifying some nonelectroactive species. For instance, a generator produces a species to be collected in the collector, but between one electrode and another this species can react with a target analyte. In this system, an indirect determination of the analyte is possible using the signal decrease at the collector.

This type of dual sensor is also helpful for studying nonelectroactive species in scanning electrochemical microscopy (SECM) experiments. For instance, one can obtain localized information on Ca^2+^ concentration during biological processes^26^ or pH changes during chemical processes such as metal electrodeposition,^27,28^ corrosion,^29,30^ and acid–base dissolution.^31,32^ For such potentiometric detection systems, a dual-electrode is often necessary as the sensing component does not help getting approaching curves. In these systems, a second component used in the amperometric mode is required to provide precise tip–substrate distance through an approach curve obtained with an appropriate redox mediator.

The SECM technique is commonly employed in two different generator–collector modes: tip generation/substrate collection (TG/SC) and substrate generation/tip collection (SG/TC).^33^ These modes are particularly useful for enzymatic studies^34–36^ and examining reaction mechanisms.^37^ The generator–collector system utilizing a dual-barrel electrode in conjunction with SECM on an insulating substrate is underexplored in the literature, especially concerning the use of collection efficiency as SECM signal to evaluate different surfaces.

This paper reports results on fabricating a dual carbon microelectrode using a dual-barrel capillary. The fabrication process employed in the present study involves isolating two carbon fibers within a double-barrel capillary using a micropipette puller. Unlike some recent works that report the fabrication of carbon dual-barrier electrodes through pyrolytic carbon deposition, which requires an inert atmosphere and a flammable gas,^35,36^ the sensor proposed in this work is constructed using commercially available carbon fibers. This represents a significantly more straightforward, safer, and more accessible approach that also eliminates the need for a vacuum in the sealing step, which can be challenging as well. The fibers are easily obtained from carbon fiber companies, eliminating the need for specialized equipment or hazardous procedures. Additionally, compared to other procedures, such as the previously mentioned one, the microdisc configuration is easier to obtain using carbon fiber, which does not change its shape during the pulling step. The electrochemical response for microdisc electrodes is also thoroughly established in the literature. We have developed an experimental protocol that allows both microelectrodes to be very close to each other, resembling a microscale of the classical rotating ring-disk configuration. The dual function of the tip components was explored through the indirect detection of thiosulfate in a generator–collector configuration coupled with an SECM technique.

## Experimental Section

### Chemicals and Materials

All solid reagents were obtained at analytical grade purity and used without further purification. All solutions were prepared using water obtained from a Nanopure Infinity System apparatus (Barnstead, Dubuque, 18.2 MΩ cm resistivity).

### Instrumentation

The produced dual-barrel microelectrode morphology was evaluated by scanning electron microscopy (SEM) images obtained using the lower secondary electron imaging method on a JEOL FESEM JSM-7401F equipment with an accelerating potential of 5 kV.

An Autolab PGSTAT128 (Eco Chemie, Utrecht, Netherlands) with data acquisition software made available by the manufacturer (Nova version 1.11, Metrohm Autolab, Utrecht, Netherlands) was used for electrochemical measurements. Experiments were performed in a conventional electrochemical cell using the fabricated dual-barrel microelectrode as working electrodes, and a Ag|AgCl|KCl_(saturated)_ as a reference electrode. The use of an auxiliary counter electrode was not required due to the very low currents achieved in this configuration.

A rotator coupled with a platinum–platinum ring-disk electrode AUT.RRDE.S (Metrohm, Autolab, Utrecht, Netherlands) was used to perform studies under controlled hydrodynamic conditions. SECM experiments were performed using an Autolab PGSTAT128 instrument coupled with a Sensolytics (Sensolytics, Bochum, Germany) SECM workstation.

### Microelectrode Fabrication

Briefly, a borosilicate theta capillary with an outer diameter of 1.5 mm and 10 cm length (Sutter Instrument Co., Novato, CA, USA) was thoroughly rinsed with acetone and dried at 60 °C for 30 min. Then, two carbon microfibers (diameter ≈ 10 μm, Solvay, Brussels, Belgium) filaments were passed through the holes of the theta capillary, one fiber per hole. The theta capillary with the fibers was pulled in a flaming/brown micropipette puller model *p*-97 (Sutter Instrument Co., Novato, CA, USA) with a temperature slightly above the melting point (RAMP + 30) and high traction (PULL = 80). Carbon fibers have high tensile strength^38^ and they are not prone to breakage during the pulling process (unlike metal wires). Hence, after pulling, the carbon fiber connecting both pipettes remained intact. Therefore, the fibers were manually cut to separate the pipettes. Afterward, the excess carbon fiber protruding from each microelectrode was trimmed. Both microelectrodes were soaked in low-viscosity instant glue (Tekbond 793; Tekbond, Brazil), which enters the cavities and grants the microelectrode seal. More details about the sealant and the pulling process are presented in the Supporting Information. To ensure the microelectrodes’ tips were flat and coplanar, the surface was polished with a homemade beveler and tested voltammetrically in hexaammineruthenium­(III) chloride ([Ru­(NH_3_)_6_]­Cl_3_) solution.

## Results and Discussion

### Dual-Barrel Microelectrode Characterization

A scanning electron microscopy (SEM) image of the tip of a typical double barrel microelectrode fabricated using the proposed method is presented in Figure 1A. The pulled glass capillary walls surrounding the carbon fibers are visible, while the two circular structures at the center of the image represent both microelectrodes. Moreover, filamentous structures, likely from the polymer used for sealing, partially cover the disk microelectrodes. The distance between the two microelectrodes was evaluated at approximately 1.3 ± 0.2 μm (*n* = 7) and the diameter of each microelectrode was found to be around 9 and 10 μm approximately. Such variation in the carbon fiber radius likely stems from the properties of the carbon fiber used in fabrication.

**Figure 1:**
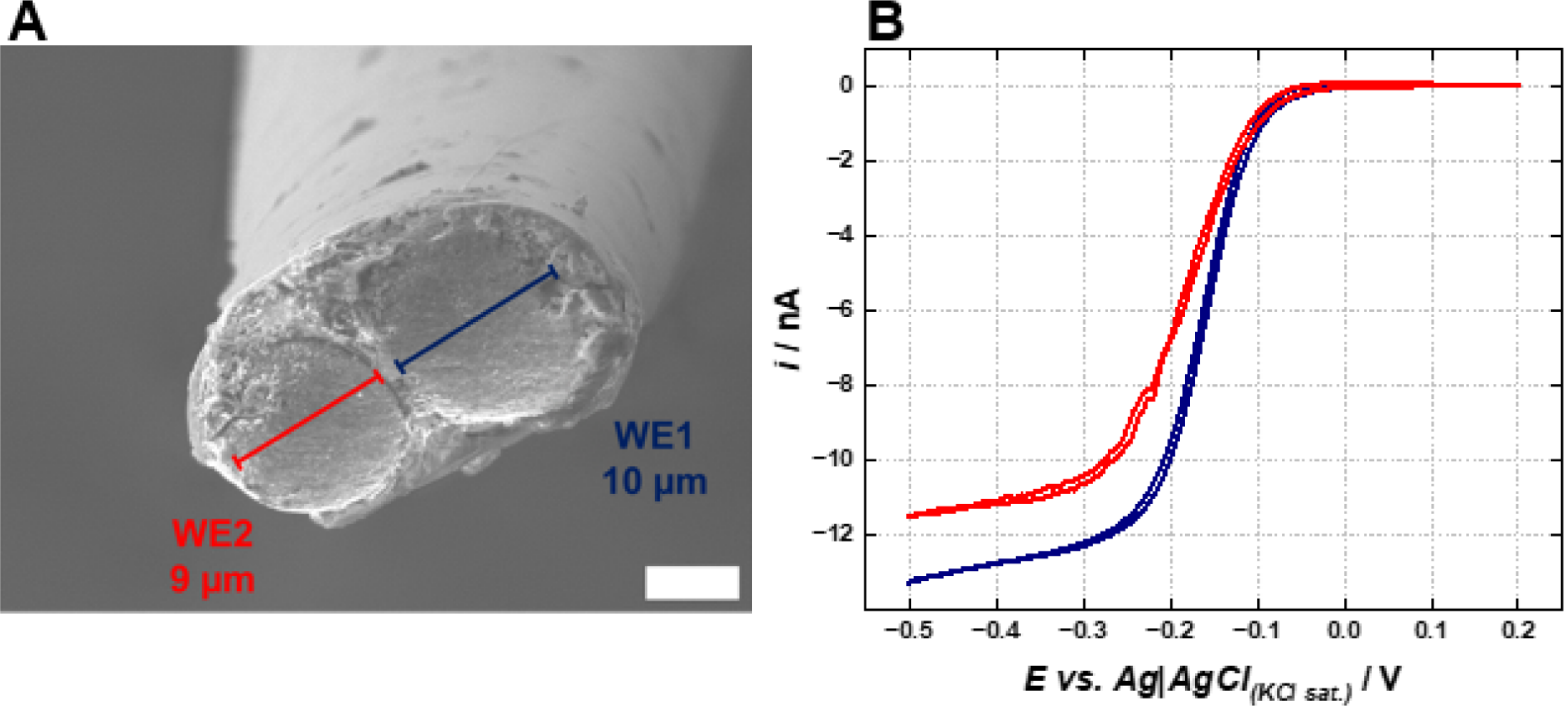
(A) Scanning electron microscopy (SEM) image of the fabricated dual-barrel carbon microelectrode. Scale bar = 5 μm. (B) Cyclic voltammograms recorded individually for both components of the dual carbon fiber microelectrode (WE1 blue; WE2 red) in a 5 mM [Ru­(NH_3_)_6_]­Cl_3_ + 0.5 M KCl solution at a scan rate of 25 mV s^–1^.

Figure 1B displays the voltammograms of both microelectrodes in the dual-barrel configuration recorded in a [Ru­(NH_3_)_6_]­Cl_3_ solution separately for each side. The voltammograms demonstrate that both microelectrodes operate independently, yielding typical sigmoidal curves with low hysteresis. Moreover, the slight variation in the limiting current for each electrode is consistent with the observation from the SEM image (Figure 1A) that one electrode is slightly smaller than the other, with relative standard deviation of 23.4% (*n* = 14), mostly possibly due to the carbon fiber thickness variation and difficult electrode polishing.

It is important to note that the proposed electrodes are not microdiscs embedded in an infinite insulating plane. Instead, they consist of two microdiscs separated by a thin insulating layer. In this configuration, for a single electrode, diffusion can occur not only from the front of the disks but also from the back of the plane (back-diffusion), leading to an almost spherical diffusion profile rather than the idealized hemispherical one, which can result in an increased limiting current, depending on the RG value. In our case, something similar is to be expected, although the back diffusion would not occur through the second microdisc part, probably resulting in a lower increase in limiting current.

Due to the proximity of the two microdisc electrodes and because radial diffusion is very efficient, the produced device presents a typical behavior of an RRDE setup without the need for forced convection. Accordingly, Figure 2 presents the generator–collector voltammograms using the fabricated dual-barrel carbon fiber microelectrode in a [Ru­(NH_3_)_6_]^3+^ solution. In this experiment, the potential of the electrode 1 (WE1, generator) is swept from 0.2 V to −0.5 V, whereas the electrode 2 (WE2, collector) is held at a constant potential (0.2 V), a value at which the species formed at the generator ([Ru­(NH_3_)_6_]^2+^) can be oxidized at the collector (eqs 1 and 2), in a process limited by diffusion.

1 $${\left[\mathrm{Ru}{\left(NH_{3}\right)}_{6}\right]}^{3+}+e^{-}\to {\left[\mathrm{Ru}{\left(NH_{3}\right)}_{6}\right]}^{2+},\left(\mathrm{at the generator}\right)$$

2 $${\left[\mathrm{Ru}{\left(NH_{3}\right)}_{6}\right]}^{2+}\to {\left[\mathrm{Ru}{\left(NH_{3}\right)}_{6}\right]}^{3+}+e^{-},\left(\mathrm{at the collector}\right)$$

**Figure 2:**
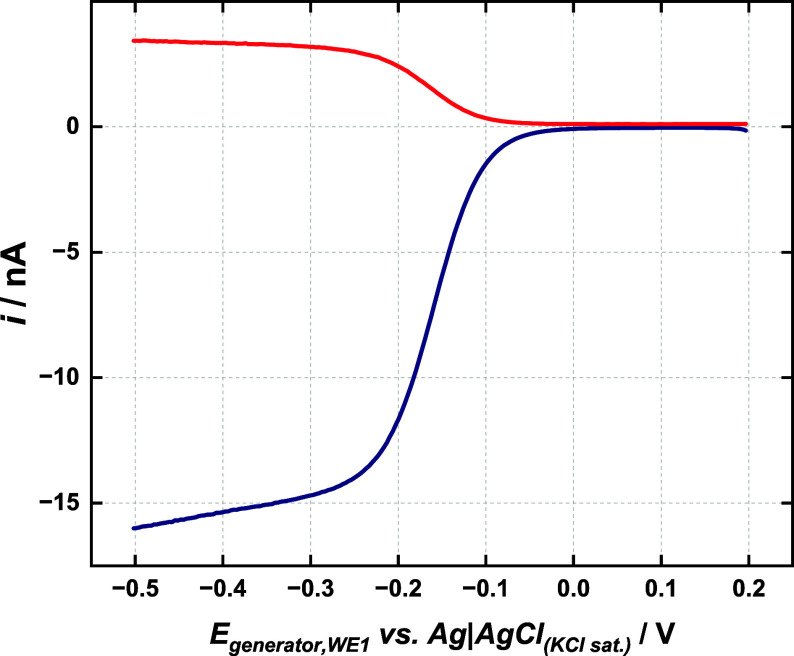
Generator (WE1 – blue line) and collector (WE2 – red line) voltammograms for the dual carbon fiber microelectrode recorded in a 5 mM [Ru­(NH_3_)_6_]­Cl_3_ + 0.5 M KCl solution. Scan rate: 25 mV s^–1^. The potential of the collector electrode was held at 0.20 V (vs Ag|AgCl_KCl sat._) during the experiment.

The current response at both microelectrodes was plotted against the generator potential (Figure 2). The collection efficiency (CE), the ratio of the collector current to the generator current, is approximately 0.24 ± 0.04 for seven fabricated devices. Hence, only a small amount of formed [Ru­(NH_3_)_6_]^2+^ reaches the collector component. This experimental CE value was compared with that obtained using a general eq (eq 9) proposed in the literature by Cutress et al.,^13^ which describes transient collection efficiencies at any given time after a potential step, where *d* and τ are dimensionless parameters that represent half gap to radius ratio and time ordinate (*τ = Dt r* ^*‑2*^, where *D* is the diffusion coefficient of the involved species, *t* is the experimental time and *r* is the electrode radius), respectively. The value found for CE using eq 9 was 0.24, which agrees well with the experimental one (0.24 ± 0.04), although this equation is optimized for dual microdiscs closely positioned in an infinite insulating substrate. Such a low CE value is expected because the material generated at the generator component diffuses to the bulk more easily than it diffuses to the collector electrode due to the electrode geometry.^21^

3 Neff=(36.64441+(d0.674)0.927)exp{−exp[−(1.2189−0.0008d2+0.3782d−0.00644d)(log(τ)−0.6206ln(2.1811d+0.3759))]}

An SECM approaching curve was recorded with the fabricated device to determine the RG (RG = *rg*/*a*, where *rg* is the radius of the microelectrode along with the surrounding insulator, and *a* is the radius of the disk-shaped microelectrode) of the microelectrode 1. Figure 3 shows the approach curve recorded against an insulating silicon substrate and the theoretical curve.^39^ The RG was obtained from the fitting as 1.7, which deviates from the geometric RG, around 1.2, obtained by SEM image. Such a deviation is expected as the geometry of this electrode greatly differs from the single disk geometry for which the used equations are defined, as the second electrode acts as an insulating surface on one side only. In other words, the electrode presents the behavior of a single disk electrode with an RG of 1.7 during the SECM approach curve, despite having different geometry and experimental RG value.

**Figure 3:**
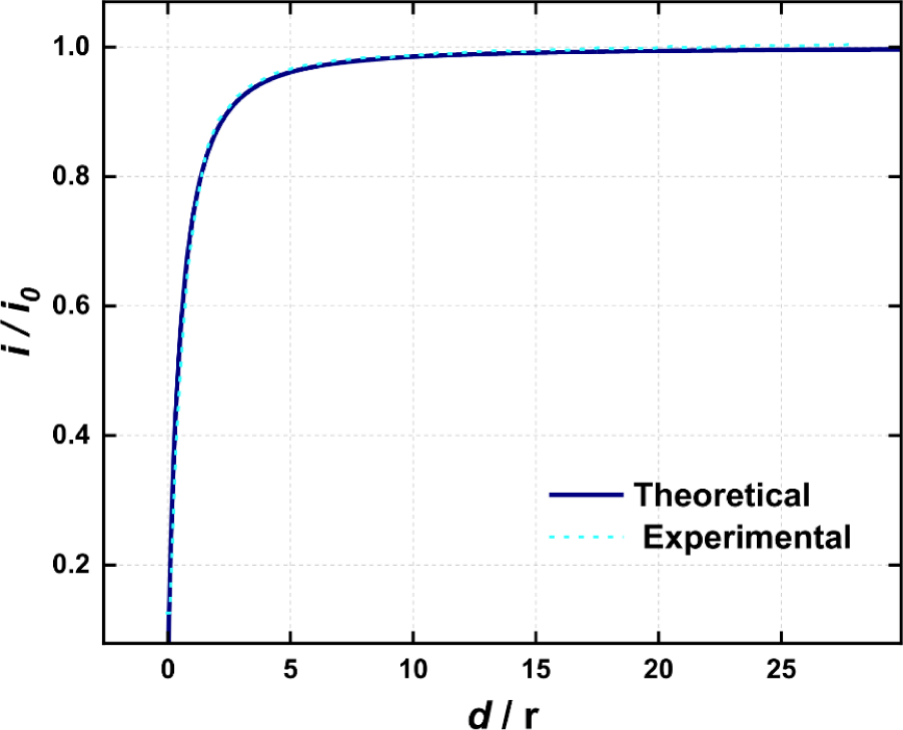
Theoretical and experimental approach curves for WE1 in the dual-barrel carbon microelectrode in a 5 mM [Ru­(NH_3_)_6_]­Cl_3_ + 0.5 M KCl solution (i_o_ = current at the bulk). E = −0.30 V. Theoretical RG = 1.7.^36^

In a further investigation, the dual microelectrode was coupled with the SECM positioning system, and an interesting increase in the collection efficiency was noticed as the electrode approached an insulating substrate. Figure 4A presents the collection efficiency in a [Ru­(NH_3_)_6_]^3+^ solution at different tip–substrate distances, while Figure 4B presents the individual generator and collector currents. In summary, the collection efficiency increases as the electrode approaches the insulating substrate up to 5 μm, from which it sharply decreases. Such behavior can be better understood by evaluating the current components (Figure 4B). Briefly, the generator electrode presents the typical SECM negative feedback (hindered diffusion) (blue line in Figure 4B), i.e., current decreases as the electrode approaches the substrate. On the other hand, the current flowing at the collector electrode (red line in Figure 4B) increases by 25 to 30% while approaching the substrate until a tip–substrate distance of 15 μm (*d* ≈ 3*a*, where *a* is the microelectrode radius). It then sharply decreases when the distance is less than 3.5 μm (*d* < 1a).

**Figure 4:**
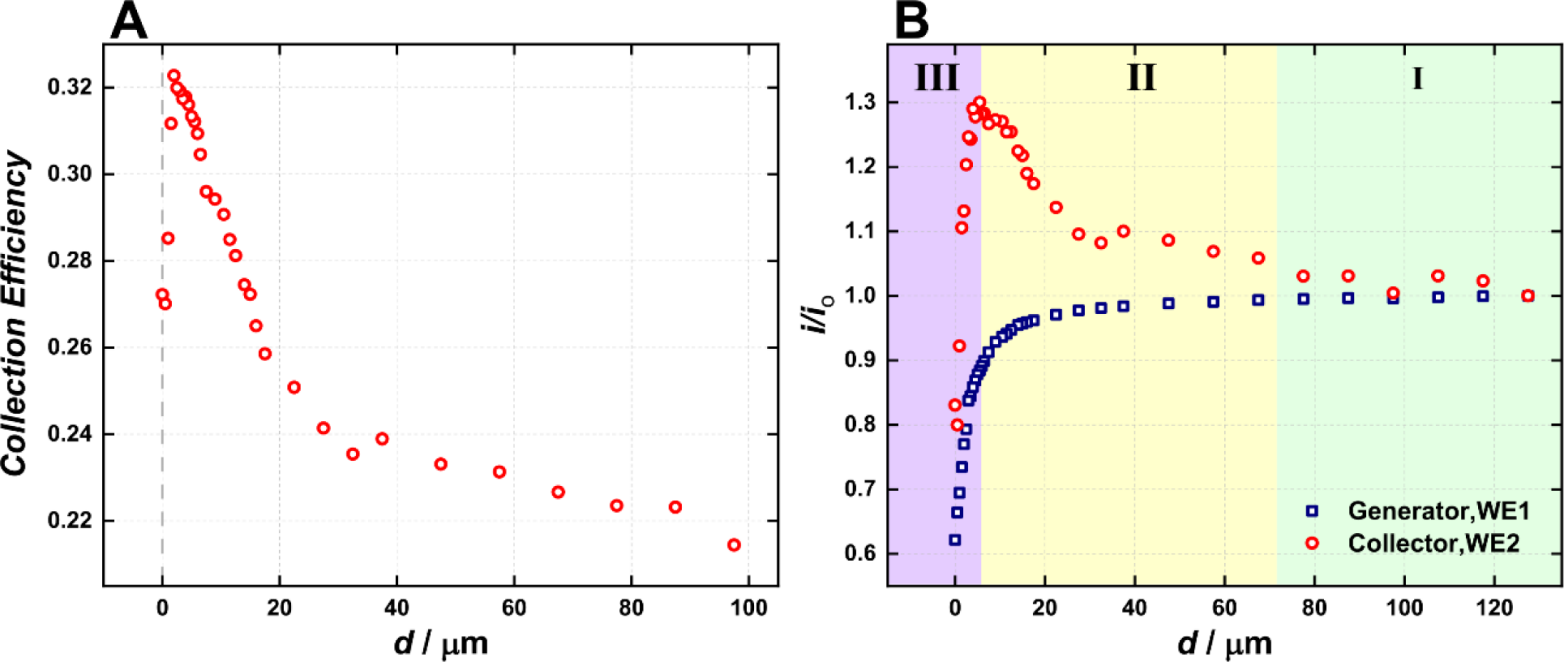
(A) Collection efficiency and (B) generator (WE1 – blue squares) and collector (WE2 - red circles) current as a function of the electrode-substrate distance to a silicon wafer substrate. Measurements were performed in a 5 mM [Ru­(NH_3_)_6_]­Cl_3_ + 0.5 M KCl solution. E_generator,WE1_ = −0.30 V and E_collector,WE2_ = 0.20 V, both measured vs Ag/AgCl_KCl Sat._ reference electrode.

Such a behavior can be explained by the species confinement when the electrode is approached to an insulating surface. Briefly, when the electrode is far from the surface, the collection efficiency depends only on the amount of [Ru­(NH_3_)_6_]^2+^ diffusing from the generator to the collector, and due to the device’s geometry, such collection efficiency is around 23% (Region I in Figure 4b and Scheme 1). However, when the electrode is moved toward the insulating surface, the generated ([Ru­(NH_3_)_6_]^2+^) is confined in a thin solution layer formed between the electrode and the substrate; hence, its diffusion to the bulk is restrained, and a relatively higher amount of [Ru­(NH_3_)_6_]^2+^ is oxidized at the collector electrode (Region II in Figure 4b and Scheme 1).

**Scheme 1:**
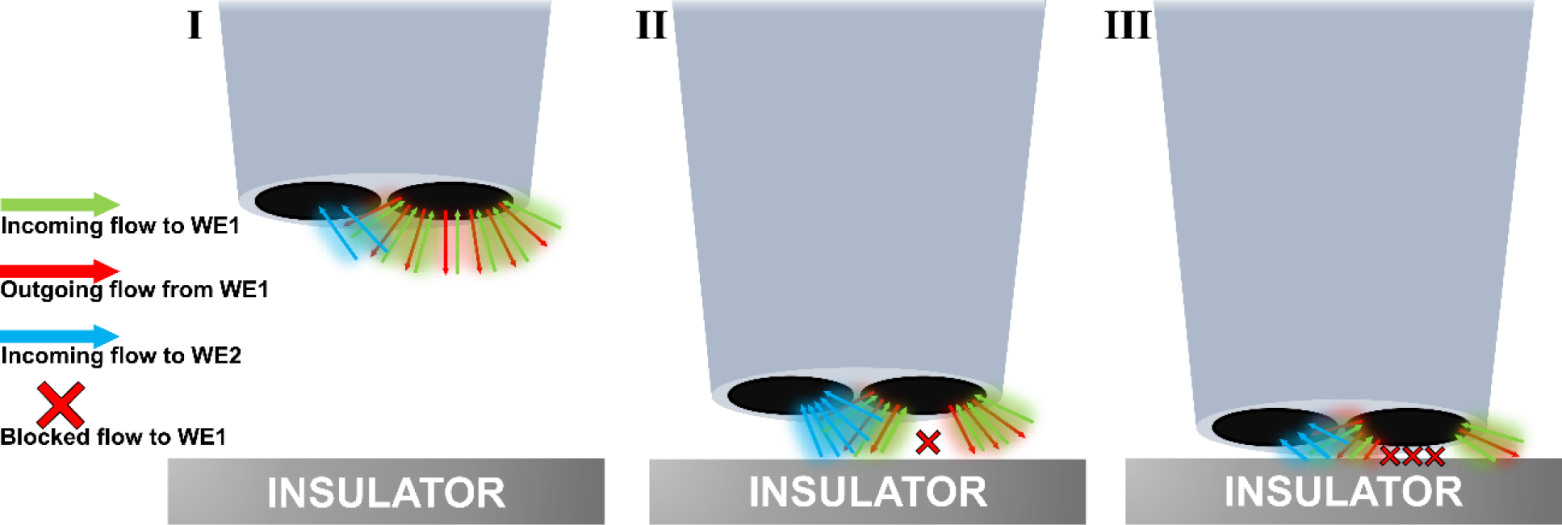
2D Illustration of the Diffusion of Species at the Dual-Barrel Microelectrode (I) Far from the Insulating Substrate, (II) Near the Insulating Substrate, and (III) Very Close to the Insulating Substrate

On the other hand, when the electrode is approached too close to the surface (tip–substrate distance less than 3.5 μm), the species formed at the generator microelectrode ([Ru­(NH_3_)_6_]^2+^) has to diffuse through an even thinner layer of solution either to the collector electrode or to the outer perimeter of the electrode. At this condition, the diffusion layer is more compressed between the electrode and the substrate, and the diffusion to the outer perimeter is favored compared to the inner perimeter and the collector electrode, thus decreasing the collection efficiency (Region III in Figure 4b and Scheme 1). The curve shape (Figure 4B, red dots) is probably dependent on the sensor geometry, and the tip–substrate distance at which the peak is noticed should vary for each electrode. Previous work in the literature^17^ shows a significantly smaller decrease in collector current at a shorter tip–substrate distance and also a decrease in collection efficiency when a generator–collector type electrode is approximated to an insulating surface.^40^ This result, however, is not easily comparable once the electrodes have a different geometry and a thinner glass wall. Also, the alignment with the substrate may affect the curve.

Experiments were also carried out with the generator collector system when approaching a conductive substrate (Figure S1). Briefly, the collection efficiency decreases as the electrode approaches a conductive substrate due to the positive feedback at the generator. Consequently, [Ru­(NH_3_)_6_]^2+^ formed at the generator is rapidly oxidized back at the conductive substrate, and such an effect is more important as the tip–substrate distance decreases. Accordingly, at the conductive substrate the collection efficiency decreases during the approaching because ***i*** the current at the generator increases due to the positive feedback and ***ii*** the current at the collector decreases as less [Ru­(NH_3_)_6_]^2+^ reaches this electrode owing to its competitive consumption by the conductive substrate. Such results agree with those reported in the literature regarding using a micro ring-disk electrode coupled to SECM in solutions containing ferrocene methanol as a redox probe.^41^

### Indirect Thiosulfate Sensing

As briefly mentioned in the Introduction, the proposed device is helpful in quantifying nonelectroactive species, such as thiosulfate, using the generator–collector device. This can be achieved using the iodide-iodine redox couple, where iodide contained in the solution is oxidized to iodine at the generator electrode (eq 9), which diffuses to the collector and is electrochemically reduced (eq 9). In the presence of thiosulfate, iodine formed in the generator can be chemically consumed during its transit to the collector (eq 9), decreasing the amount of iodine reaching the collector electrode and thus decreasing the recorded current. Therefore, the thiosulfate is detected indirectly through the decrease in the collector current.

4 $$2I^{-}\to I_{2}+2e^{-},\left(\mathrm{at the generator}\right)$$

5 $$I_{2}+2e^{-}\to 2I^{-},\left(\mathrm{at the collector}\right)$$

6 $$I_{2}+2S_{2}{O_{3}}^{2-}⇌S_{4}{O_{6}}^{2-}+2I^{-},\left(\mathrm{in the solution}\right)$$

Similar procedures have been already described in the literature in experiments with RRDE,^42^ interdigitated array microelectrodes,^43^ and a thin-layered dual-band electrochemical cell.^4^

The formation of an iodine film on the electrode surface during the electrochemical oxidation of iodide occurs due to the low solubility of I_2_ in water (∼1.3 mM). The adsorption of iodine at electrode surfaces is well-known,^44–47^ and such a process has been proved using the electrochemical quartz crystal microbalance (EQCM) and the RRDE technique. Therefore, preliminary experiments were performed to establish the electrochemistry of iodide at the fabricated dual-carbon microelectrode. Accordingly, Figure S2 shows CVs recorded in a 2 mM iodide solution at different scan rates using the WE1 of the fabricated device. Experiments were carried out in an acidic medium to avoid the disproportionation of iodine, and the upper potential limit was fixed at 1.0 V, as electrogenerated iodine can be further oxidized to iodate at more positive potentials.^48^ The typical sigmoidal voltammogram is noticed at lower scan rates, whereas a cathodic component corresponding to the electroreduction of iodine is observed at higher scan rates. The presence of a cathodic component in a CV recorded with a small radius microelectrode (*r* = 5 μm) is unexpected because radial diffusion would prevail even at 200 mV s^–1^ (note that there is no change in the anodic component independent of the scan rate). Such surprising behavior may be attributable to the iodine adsorption at the electrode surface, i.e., the product does not diffuse from the electrode surface to the bulk solution, resulting in the cathodic component. Iodine can be dissolved by iodide according to the following eq 9 ^49^ and such an event is time-dependent.^44^

7 $$I_{2}+I^{-}⇌I_{3},K=2.3\times 10^{-4}L{\mathrm{mol}}^{-1},\left(\mathrm{at}\;25\;{\;}^{{}^{\circ}}C\right)$$

However, this is not expected because iodide is completely depleted at the electrode surface at sufficiently positive potentials,^50^ but the formation of triiodide through the reaction between iodide and iodine is plausible at conditions where iodide can be found at the electrode surface, i.e., at potentials where the iodide surface concentration remains high and triiodide is the main product (I < I_L_).^51^ Hence, the amount of iodine dissolved by iodide during the reverse scan and at potentials less positive than 0.6 V (where the concentration of iodide at the surface is not zero) decreases as the time window decreases (increased scan rate), leading to the cathodic component.

An additional experiment was carried out to corroborate such an assumption. As the iodine dissolution is time-dependent, a rest time at which the microelectrode was not biased was included before the reverse scan. Experiments were carried out at different rest times, and Figure S3 shows the results. As expected, iodine accumulated on the carbon fiber surface is assumed to be dissolved by iodide, and a decrease in the cathodic current (dependent on the amount of adsorbed iodine) was noticed at higher rest times. Such a result demonstrated that a procedure is required to clean the electrode surface after each CV, and this was accomplished by keeping the potential at 0 V for 15 s before each new measurement to promote iodine electroreduction.

Figure 5 shows generator–collector voltammograms recorded with the dual carbon fiber microelectrode in iodide solution. The collection efficiency value found in such an experiment was similar to that found in the [Ru­(NH_3_)_6_]^3+^ /[Ru­(NH_3_)_6_]^2+^ system. This demonstrates that iodine does not accumulate on the generator electrode in this experimental condition. Additional generator–collector experiments in iodide solution at the same concentration were carried out using RRDE voltammetry and a CE value close to that obtained in the [Ru­(NH_3_)_6_]^3+^ /[Ru­(NH_3_)_6_]^2+^ system was obtained, reinforcing the conclusion regarding the absence of iodine adsorption.

**Figure 5:**
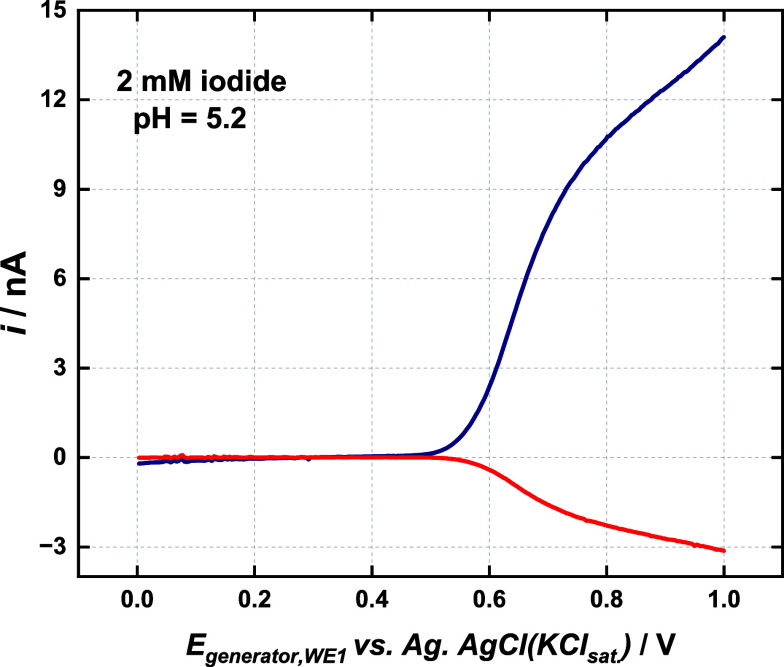
Generator (WE1 – blue line) and collector (WE2 – red line) voltammograms for the dual carbon fiber microelectrode recorded in a 2 mM iodide pH = 5.2. Scan rate = 10 mV s^–1^. The potential of the collector electrode was held at 0.0 V vs Ag/AgCl_KCl Sat_.

A calibration plot for thiosulfate was obtained using the collector current as a signal. Figure 6A presents the voltammograms corresponding to the collector current as a function of the potential applied to the generator at different thiosulfate concentrations, and Figure 6B shows the calibration plot. Such experiment was performed at pH around 5 because thiosulfate is decomposed in highly acidic conditions and iodine is not stable in an alkaline medium, as it is transformed to hypoiodite (IO^–^). A narrow linear range extending from 10 μM up to 200 μM (i (nA) = 4.94 × 10^–3^ −2.87; R^2^ = 0.999) was noticed, and the detection limit for such a system was calculated as 6 μM, by 3σ/*s*. (where σ is the standard deviation of the intercept and *s* is the slope) On the other hand, a nonlinear range was observed for higher thiosulfate concentrations. For comparison, experiments were repeated using an RRDE. The mass transfer coefficient, *k*_*t*_, for diffusion to a disk microelectrode is given by eq 9 and for a rotating disk electrode, by eq 9.^52^

8 $$k_{t}=4D/\pi a$$

9 $$k_{t}=0.62D^{2/3}\upsilon^{-1/6}\omega^{1/2}$$

where *D* is the diffusion coefficient of the redox probe, *a* is the disk radius, υ is the kinematic viscosity, and ω is the rotation rate. A mass transport coefficient of 0.049 cm s^–1^ is calculated for a 5 μm radius microelectrode, and to achieve the same mass transport coefficient, the RRDE should be rotated at 23,000 rpm, assuming a *D* value of 1.76 × 10^–5^ cm^2^ s^–1^ ^53^ and kinematic viscosity of 0.0093 cm^2^ s^–1^.^54^ Such an experimental condition is unfeasible because of the turbulence and voltammograms were then recorded with an RRDE at 4000 rpm. This resembles a 12 μm radius microelectrode and not the fabricated 5 μm radius microelectrode, but a rough comparison can be made. Results shown in Figure S4 demonstrate that the behavior seen in Figure 6B is consistent wherever the iodine transport from the generator to the collector electrode is achieved through radial diffusion (dual microelectrode) or forced convection (RRDE).

**Figure 6:**
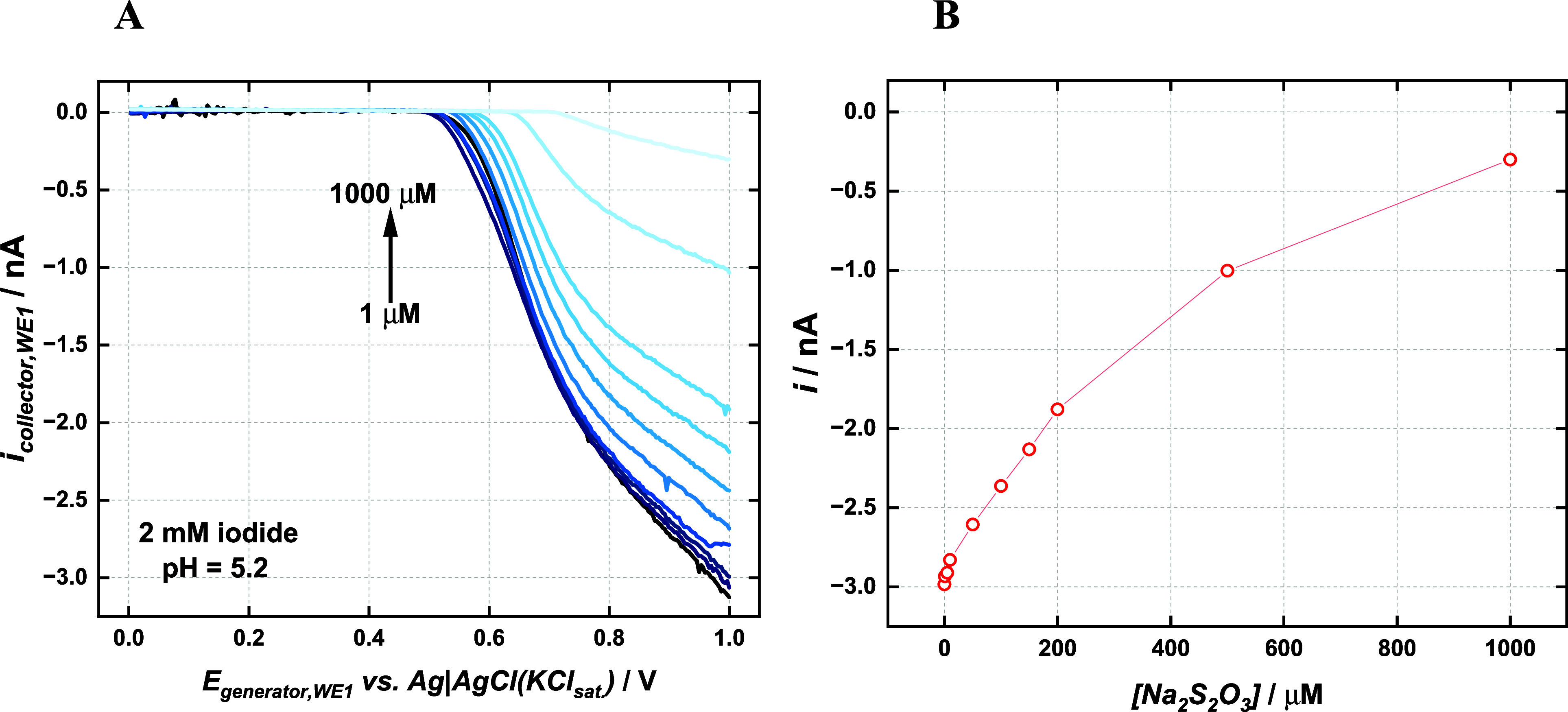
(A) Voltammograms recorded with the dual carbon microelectrode in a 2 mM iodide solution at pH = 5.2 and different thiosulfate concentrations. Scan rate= 10 mV s^–1^. (B) Calibration plot using current values measured at the collector electrode at 0.0 V.

The amount of iodine that reaches the collector electrode in the dual carbon microelectrode depends on the generator–collector distance. In a solution containing thiosulfate, another variable is added: the rate of iodine consumption by thiosulfate. Therefore, time is another critical factor in defining current at the collector besides the geometric parameter. Additional experiments were performed using the RRDE to shed light on the behavior shown in Figure 6B. In this configuration, the transit time between the generator and the collector can be more easily modulated than changing the distance between both microelectrodes in the dual configuration. Accordingly, Figure S5 displays the collection efficiency as a function of the square root of the rotation rate in an iodide solution. In the absence of thiosulfate, no significant change in collection efficiency is observed, as expected, since the rate at which iodide reaches the generator electrode is equal to the rate at which iodine reaches the collector electrode at each rotation rate. Introducing thiosulfate adds a new variable to the system: the kinetics of the chemical reaction between thiosulfate and iodine. Although more iodine is delivered to the collector electrode under higher rotation rates, increased convection also enhances the thiosulfate transport from the bulk solution to the reaction layer. This may lead to an increasingly extensive consumption of iodine and decreased collection efficiency. Thus, the reaction rate becomes a critical variable in this indirect detection system.

## Conclusions

This work demonstrates that a dual-carbon microelectrode can be properly and easily fabricated using a dual-barrel theta capillary. Because both microelectrodes can be closely arranged in the device, generator–collector experiments, which involve the generation of a species at one electrode and its subsequent collection at another, are easily designed. In such a configuration, the device was successfully explored to get indirect analytical information on the thiosulfate concentration through changes in the collector current in the iodide/iodine system. Furthermore, it is possible to enhance the collection factor for an individual device by approaching it to an insulating substrate using SECM.

High spatial resolution measurements with biosensors in biological medium containing possible interfering compounds can be performed exploiting the “sentinel” (or null-electrode) concept.^55,56^ The sentinel sensor does not include the sensing element (typically an enzyme) and ensures that current changes are only due to changes in the analyte concentration. Because both components of the proposed device can be closely arranged, one of the elements can be employed as the sentinel electrode for localized measurements in complex environments.

The fabricated device’s potential is also noteworthy, particularly in situations requiring a single detection system using multiple miniaturized sensors. For heterogeneous systems, spatially resolved measurements require devices containing independent sensors and assemblies with micrometric dimensions to examine heterogeneities. With its closely arranged components and micrometer-dimension range capillaries, the proposed device holds promise for performing measurements in biological samples without significant tissue damage, opening up a wide range of interesting applications.

## Supplementary Material



## Notes

Pulling process parameters description. Chemical specifications of sealant. Complementary experiments graphs (PDF)
