# Supplementary material for: A Dual-Barrel Carbon Fiber Microelectrode for Generator–Collector Experiments
Source: ACS Omega. 2025 Aug 7;10(32):36550–8. doi: 10.1021/acsomega.5c05321 (PMC12368688; doi:10.1021/acsomega.5c05321)
Supplement: Supplementary file 1 [file ao5c05321_si_001.pdf]

Supplementary information

A dual-barrel carbon fiber microelectrode for generator-collector experiments

Paula C. Falcoswki[1], Douglas P.M[1]. Saraiva, Nicolas A. Ishiki[2], Edson A. Ticianelli[3], Mauro Bertotti[1]*

[1]Universidade de São Paulo, Departamento de Química Fundamental, Instituto de Química, São Paulo, Brasil

[2]Université Paris Cité, ITODYS, CNRS, Paris, France

[3]Universidade de São Paulo, Departamento de Físico Química, Instituto de Química de São Carlos, C.P. 780, São Carlos, Brasil

1. Pulling process

The pulling step was conducted in the absence of vacuum by the pulling machine P-97 (Sutter Instrument Co., Novato, CA, USA) with the following parameters:

Heat: ramp +30; Pull: 80; Vel: 40; Time :200

It was pulled over 40 microelectrodes and analyzed by an optical microscope, although not all of them achieved the electrochemical test step due to the fragile nature of pulled micropipettes. No bubbles between the microfiber and glass or fiber rupture was observed during the process.

On average, one capillary leads to one dual microelectrode due to the fact that the carbon fiber can slide out through one of the micropipettes if it is not well sealed.

2. Chemical specifications of sealant

As sealant, we successfully employed both a low-viscosity epoxy resin (Bisphenol A diglycidyl ether, Avipol 2004; viscosity: around 60 cP; Avipol, Brazil) and an acrylate-based superglue (Tekbond 793; viscosity: 80–120 cP; Tekbond, Brazil). The ethylcyanoacrylate (PCA) superglue, as presented in this work, is often described as biodegradable; however, it can release toxic byproducts upon contact with biological fluids and is therefore not approved for human applications. In contrast, the epoxy resin (BADGE: Bisphenol A diglycidyl ether) is considered bio-inert, as bisphenol A is not released after full curing due to the extensive polymer crosslinking.

3. Complementary experiments

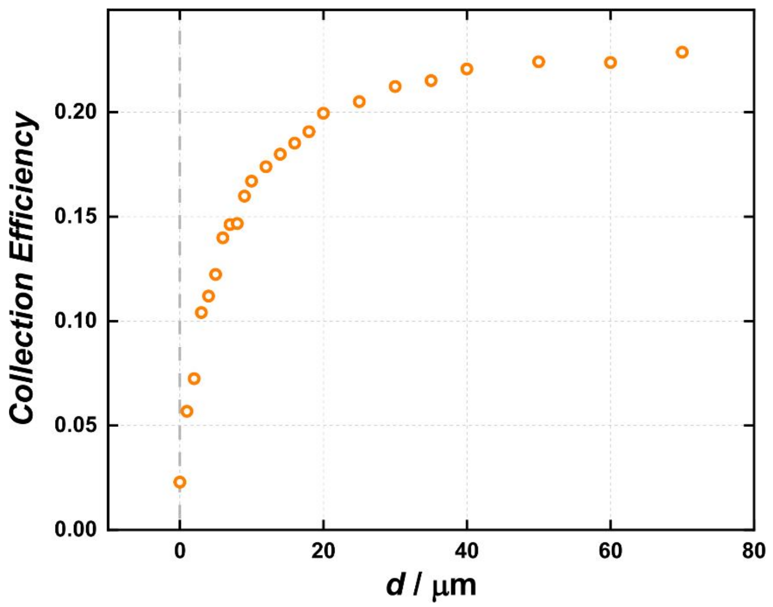

Figure S1 – *Collection efficiency as a function of the electrode-substrate distance to an Au substrate. Measurements were performed in a 5 mM $[Ru(NH_3)_6]Cl_3$ + 0.5 M KCl solution. $E_{generator,WE1}$ =-0.30 V and $E_{collector, WE2}$ = 0.20 V, both measured vs. Ag/$AgCl_{KCl\ Sat.}$ reference electrode.*

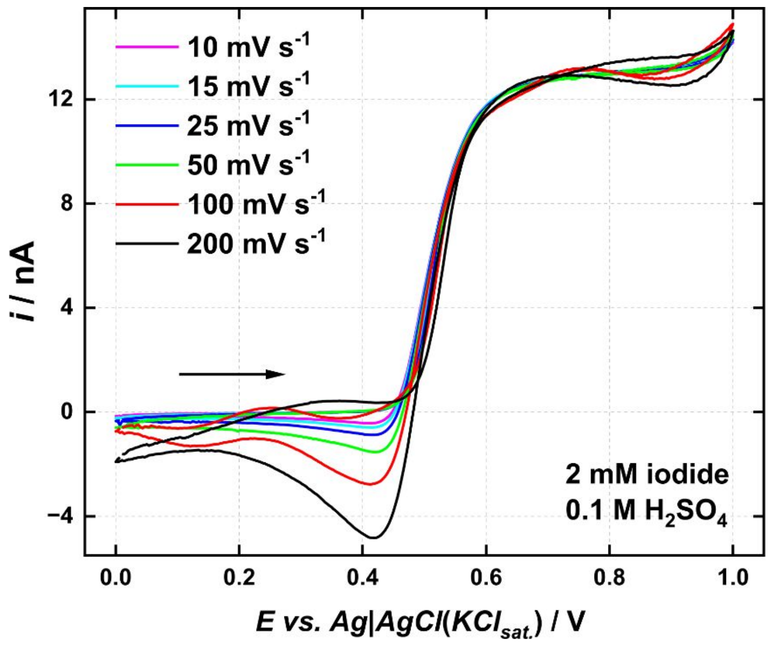

Figure S2 – *Cyclic voltammograms recorded with WE1 of the dual carbon fiber microelectrode in a 2 mM iodide + 0.1 M H_2SO_4 solution at different scan rates.*

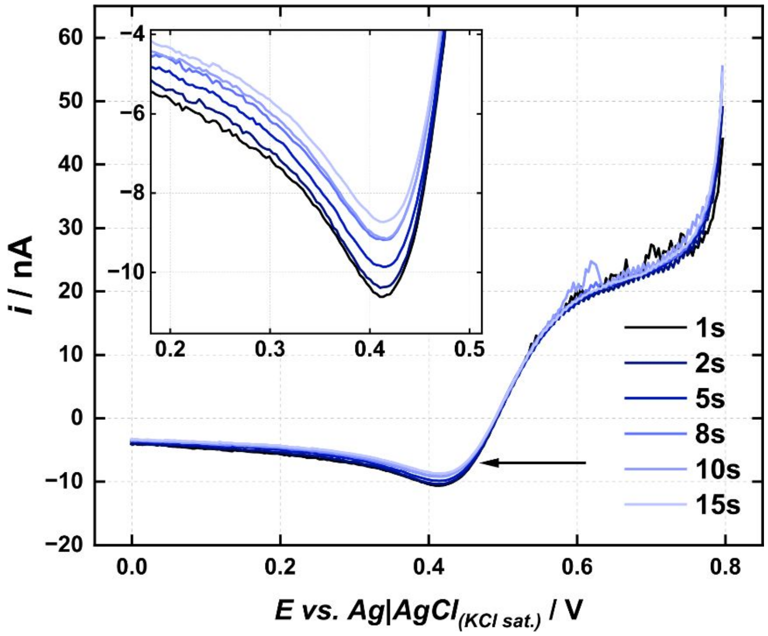

Figure S3 – *Linear Scan Voltammetry recorded with WE1 of the dual carbon fiber microelectrode in a 2 mM iodide + 0.1 M H_2SO_4 solution at different waiting times of electrode depolarization after the forward scan. Scan rate: 10 mV/s.*

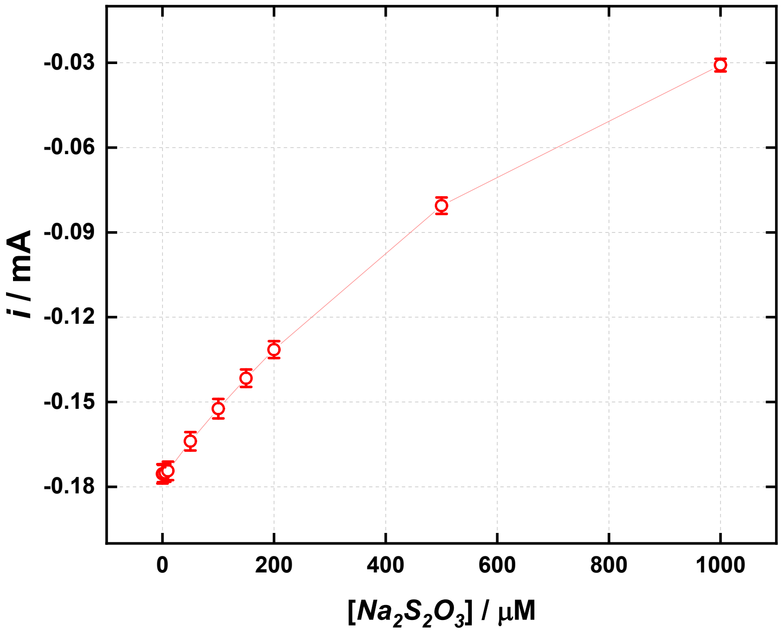

Figure S4 – *Calibration plot corresponding to the indirect quantification of thiosulfate using the iodide/iodine system using an RRDE Pt-Pt electrode. Ring electrode held at 0.0 V in a 2 mM iodide solution + H_2SO_4 at pH 5.2. Scan rate= 10 mV s^{-1}. RRDE rotation speed: 4000 rpm. Measurements carried out in triplicate.*

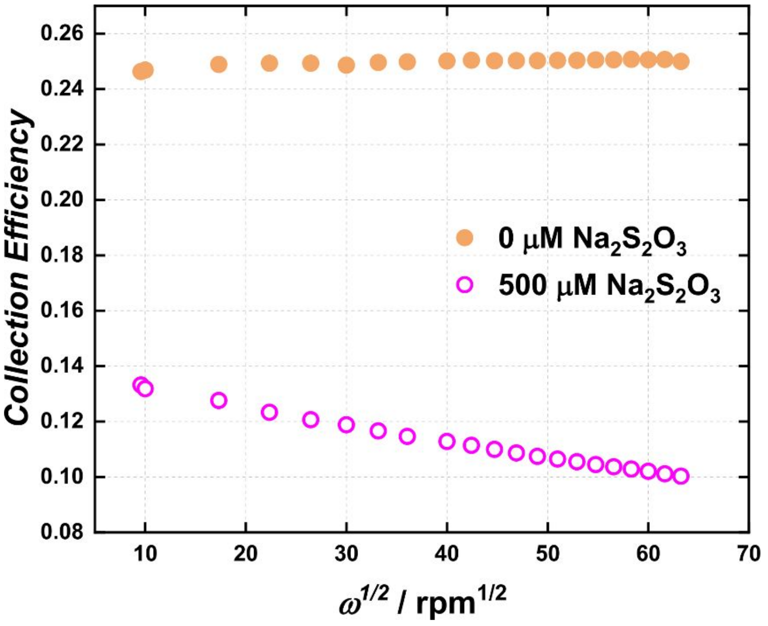

Figure S5 – *Collection efficiency values as a function of the square root of the rotation rate in experiments performed with a platinum-platinum ring-disk electrode in a 2 mM KI + 0.1 M Na_2SO_4 solution at pH=5.2, in the presence and absence of thiosulfate.* $E_{collector} = 0$ *V.*
